# MacAB-TolC Contributes to the Development of *Acinetobacter baumannii* Biofilm at the Solid–Liquid Interface

**DOI:** 10.3389/fmicb.2021.785161

**Published:** 2022-01-13

**Authors:** Brandon Robin, Marion Nicol, Hung Le, Ali Tahrioui, Annick Schaumann, Jean-Baptiste Vuillemenot, Delphine Vergoz, Olivier Lesouhaitier, Thierry Jouenne, Julie Hardouin, Anaïs Potron, Valérie Perrot, Emmanuelle Dé

**Affiliations:** ^1^Normandie Univ, UNIROUEN, INSA Rouen, CNRS, Polymers, Biopolymers, Surfaces Laboratory, Rouen, France; ^2^Normandie Univ, UNIROUEN, LMSM EA4312, Evreux, France; ^3^PISSARO Proteomic Facility, IRIB, Mont-Saint-Aignan, France; ^4^UMR 6249 Chrono-Environnement, CNRS-Université de Bourgogne/Franche-Comté, Besançon, France

**Keywords:** solid–liquid interface, biofilm, efflux pump, eDNA, envelop stress response

## Abstract

*Acinetobacter baumannii* has emerged as one of the most problematic bacterial pathogens responsible for hospital-acquired and community infections worldwide. Besides its high capacity to acquire antibiotic resistance mechanisms, it also presents high adhesion abilities on inert and living surfaces leading to biofilm development. This lifestyle confers additional protection against various treatments and allows it to persist for long periods in various hospital niches. Due to their remarkable antimicrobial tolerance, *A. baumannii* biofilms are difficult to control and ultimately eradicate. Further insights into the mechanism of biofilm development will help to overcome this challenge and to develop novel antibiofilm strategies. To unravel critical determinants of this sessile lifestyle, the proteomic profiles of two *A. baumannii* strains (ATTC17978 and SDF) grown in planktonic stationary phase or in mature solid–liquid (S-L) biofilm were compared using a semiquantitative proteomic study. Of interest, among the 69 common proteins determinants accumulated in the two strains at the S-L interface, we sorted out the MacAB-TolC system. This tripartite efflux pump played a role in *A. baumannii* biofilm formation as demonstrated by using Δ*macAB-tolC* deletion mutant. Complementary approaches allowed us to get an overview of the impact of *macAB-tolC* deletion in *A. baumannii* physiology. Indeed, this efflux pump appeared to be involved in the envelope stress response occurring in mature biofilm. It contributes to maintain wild type (WT) membrane rigidity and provides tolerance to high osmolarity conditions. In addition, this system is probably involved in the maintenance of iron and sulfur homeostasis. MacAB-TolC might help this pathogen face and adapt to deleterious conditions occurring in mature biofilms. Increasing our knowledge of *A. baumannii* biofilm formation will undoubtedly help us develop new therapeutic strategies to tackle this emerging threat to human health.

## 1. Introduction

Over the last decades, *Acinetobacter baumannii* has emerged as one of the most problematic opportunistic pathogens involved in hospital-acquired infections and community infections worldwide (Lin and Lan, 2014). The pathogenicity of this member of the ESKAPE group of bacterial pathogens (*Enterococcus faecium*, *Staphylococcus aureus*, *Klebsiella pneumoniae*, *A. baumannii*, *Pseudomonas aeruginosa*, and *Enterobacter* spp.) (Boucher et al., 2009) and its success as an infective agent appear to be related to multiple factors, and especially its ability to form biofilms. Indeed, its high capacity to acquire antibiotic resistance mechanisms has led to the increasing occurrence of outbreaks of infection involving multi- or pan-drug-resistant *A. baumannii* (Lee et al., 2017; Nasr, 2020). Furthermore, it also presents remarkable adhesion abilities on inert and leaving surfaces, leading to biofilm development that allows it to survive desiccation (Gayoso et al., 2014), oxidative stress (Soares et al., 2010), or disinfectants (Peleg et al., 2008; Harding et al., 2018) and hence to persist for long periods in various hospital environments. This concerning public health threat was therefore ranked on the global priority pathogens list established by the World Health Organization (WHO) for which there is an urgent need for new antibiotic development.

Biofilms are structural communities of interface-associated bacteria organized as microcolonies embedded within a complex hydrated polymeric matrix composed of extracellular polymeric substances (EPSs), such as exopolysaccharides, proteins, nucleic acids, and other compounds (Monds and O’Toole, 2009). The regulatory process of biofilm formation is highly dynamic and influenced by environmental factors that allow the transition between free-floating cells and biofilm lifestyles. The sessile growth mode provides clear ecological and physiological advantages to microorganisms that inherently benefit of protection against adverse environments, host immune system clearance, antibiotics, and other antimicrobial agents and protection from starvation through carbon storage (Yan and Bassler, 2019; Zhang et al., 2020). In addition, bacterial biofilm appears also to be an ideal environment for the horizontal exchange of genetic material between microorganisms through genetic mutations and rearrangements and also integration of determinants carried by mobile genetic elements, thus reinforcing bacterial genetic plasticity (Soucy et al., 2015). It is now well established that bacterial biofilm is involved in lots of infectious diseases and in a variety of medical device-related infections (Zhang et al., 2020). Indeed, the pathogenic potential of sessile microorganisms is much higher than the one of planktonic cells.

The ability of *A. baumannii* to form a biofilm is one of the leading mechanisms that has largely contributed to its success as a human pathogen. This Gram-negative bacterium may cause severe nosocomial infections including hospital-acquired and ventilator-associated pneumonia, bacteremia, endocarditis, skin and soft tissue infections, urinary tract infections, or meningitis (Peleg et al., 2008; Nasr, 2020). Biofilms are commonly referred to as solid-attached structures, but they can develop on a wide variety of interfaces including solid–liquid (S-L), air–liquid (A-L), liquid–liquid, or air–solid interfaces. *A. baumannii* biofilms grow at S-L interfaces, e.g., between a biological or an abiotic surface and an aqueous medium, but this organism has also been characterized for its ability to develop A-L interface biofilms, also known as pellicles, which constitute more complex structures than classical surface-attached biofilms in terms of development, level of organization, and mechanics (Marti et al., 2011b). Some of our investigations have revealed that *Acinetobacter* species forming pellicles, such as *A. baumannii* and *A. nosocomialis*, are those mainly involved in nosocomial infections, suggesting a correlation between this sedentary lifestyle and bacterial pathogenicity (Marti et al., 2011a; Kentache et al., 2017).

Despite the abundant literature on biofilm lifestyles and their widespread distribution in diseases, some issues remain unclear. Owing to their increasing resilience to antimicrobial treatments, *A. baumannii* biofilms are difficult to control and ultimately eradicate. Our understanding of this pathogen is that biofilm formation rather facilitates and/or prolongs its survival in harsh conditions likely by adopting a “persist and resist” strategy as previously proposed (Harding et al., 2018). Therefore, it is urgently needed to decipher mechanisms involved in *A. baumannii* biofilm formation and thus to identify key determinants that can be potential targets, aiming at developing novel anti-biofilm strategies. In this context, efflux pumps constitute critical determinants of this sessile lifestyle and have emerged as promising targets, as their inhibition may allow to fight pathogens at various levels, antibiotic resistance, but also biofilm formation. Indeed, in some bacterial species, such as *Escherichia coli*, tripartite efflux pumps have been previously reported to be involved in biofilm formation (Alav et al., 2018). In *A. baumannii*, it was envisaged that the Pmt [putative major facilitator superfamily (MFS) transporter-like] protein could be associated with the release of eDNA and adhesion on biotic and abiotic surfaces (Sahu et al., 2012). Another MFS transporter, AbeF, involved in fosfomycin efflux, was also proposed to participate in the secretion of biofilm matrix (Sharma et al., 2017). The contribution of resistance-nodulation-division (RND)-efflux pumps, like AdeABC, to this growth mode was also demonstrated but especially in terms of adhesion (Richmond et al., 2016). Finally, deletion of the efflux pump genes *emrA/emrB* resulted in a decrease of biofilm formation in *A. baumannii*, even though their precise roles remained to be clarified (Lin et al., 2020).

In the current study, we have compared the biofilm-forming ability of two strains of *A. baumannii* harboring specific features. We used the SDF strain that interestingly produces an abundant biofilm, but not pellicle, without presenting the main classical determinants associated with virulence of biofilm (such as Csu pili, PgaABCD, and type IV pili) (Antunes et al., 2011; Eijkelkamp et al., 2014), and the *A. baumannii* ATCC 17978 strain as a reference strain. The proteome profiles of bacteria grown in planktonic stationary phase with those of bacteria grown in mature S-L biofilm were compared using a proteomic semiquantitative study. This analysis highlights adhesins that could contribute to initiation and development of the SDF biofilm. Of interest, among the 69 common protein determinants accumulated by the two *A. baumannii* strains at the S-L interface, we sorted out the MacAB-TolC system. This pump has been reported to actively extrude various substrates, including macrolide antibiotics and virulence factors in *E. coli* and other Gram-negative bacteria. It was also involved in the transport of outer membrane glycolipids, lipopeptides, and protoporphyrin (reviewed in Fitzpatrick et al., 2017). Interestingly, this tripartite efflux pump appears to be a noteworthy determinant of *A. baumannii* mature biofilms as demonstrated by using *macAB-tolC* deletion mutant. Complementary approaches allowed us to suggest its contribution to iron and sulfur homeostasis and to demonstrate its involvement in cell wall rigidity and osmotic protection.

## 2. Materials and Methods

### 2.1 Bacterial Strains and Growth Conditions

Strains and plasmids used in this study are listed in Supplementary Table 1. *A. baumannii* SDF strain was selected based on its failure to form pellicle and the lack of the main classically defined determinants of biofilm (Fournier et al., 2006). The ATCC 17978 strain, lacking the pAB3 plasmid (pAB3-) as checked by PCR amplification and sulfamethoxazole/trimethoprim (SXT) susceptibility testing (Weber et al., 2015), was chosen because of its high capacity to form biofilms compared to ATCC 17978 pAB3+ strain. The SDF strain was grown in Luria Bertani medium (LB, Difco; Antunes et al., 2011). ATCC 17978 and its derivative strains were grown in Mueller–Hinton broth (MHB, Difco). The mutant strains complemented with pWH1266 (ΔMac_e) or pWH1266::*macAB-tolC* (ΔMac_c) were selected on MHB supplemented with 10 μg/ml ticarcillin. *E. coli* DH5α (pCR-Blunt) and CC118λpir (pKNG101) were selected on MHB containing 50 μg/ml kanamycin and 50 μg/ml streptomycin, respectively.

### 2.2. Mutant and Complemented Strain Construction

Deletion mutant was constructed from *A. baumannii* ATCC 17978 using overlapping PCRs and recombination events according to the protocol of Richardot et al. (2016). Briefly, the 5′ region of *tolC* gene (ABYAL0571, 703 bp) and the 3′ region of *macA* gene (ABYAL0574, 816 bp) were amplified by PCR with specific primers (Supplementary Table 2). The resulting PCR products were used as templates for overlapping PCRs to generate the mutagenic DNA insert Δ*macAB-tolC*. The insert was cloned into pCR-Blunt plasmid, then digested with *Bam*HI/*Apa*I. The generated fragment was subcloned into pKNG101, then the resulting plasmid pKNG101::Δ*macAB-tolC* was transferred into *E. coli* CC118λpir. The suicide vector was introduced into *A. baumannii* strain by triparental mattings using *E. coli* HB101 (pRK2013) helper strain. *A. baumannii* with pKNG101::Δ*macAB-tolC* was selected on MH agar supplemented with 800 μg/ml streptomycin, and *E. coli* was counterselected with 30 μg/ml chloramphenicol. Suicide vector pKNG101 with the *macAB-tolC* genes was excised by selection on M9 medium agar plates supplemented with 5% sucrose. The plasmid loss was confirmed by negative selection on MH agar with 800 μg/ml streptomycin, and the deletion of 4,524 bp was checked by PCR and sequencing (Supplementary Table 2). The entire operon *macAB-tolC* from *A. baumannii* ATCC 17978 was amplified using specific primers containing a complemented sequence from the expression vector pWH1266 (Supplementary Table 2). The plasmid pWH1266 was linearized with *HindIII* enzyme and then reassembled with the *macAB-tolC* PCR product using NEBuilder DNA Hifi Assembly kit (New England Biolabs). The resulting plasmid pWH1266::*macAB-tolC* was transferred into *E. coli* DH5a by transformation and then into Δ*Mac* strain by electroporation.

### 2.3. Proteomic Analyses of Planktonic and Sessile Bacterial Cultures

Biofilms were grown on 30 g of glass wool in 800 ml of rich medium using 10^7^ [Colony Forming Unit (CFU)/ml] as an inoculum (Crouzet et al., 2017). They were incubated at 37°C for 4 days with slight shaking (90 rpm) to avoid pellicle formation. Then, glass wool was washed three times with phosphate buffer saline (PBS) to remove unattached cells. Biofilm bacteria were recovered from glass wool by vigorous shaking with 30 g of glass beads and a subsequent centrifugation (6,000 *× g*, 15 min, 4°C). One-day-old planktonic cultures were performed similarly but with shaking at 140 rpm and without glass wool. Total protein extraction from planktonic and biofilm cells was performed as already described (Kentache et al., 2017). Protein samples were prepared at least in biological triplicate for each condition. Then, enzymatic digestion of protein extracts and quantitative analysis by mass spectrometry analyses were performed according to Kentache et al. (2017). Protein abundances in the wild type (WT) and ΔMac were compared using Progenesis LC-MS software for protein quantification. False discovery rates (FDRs) were calculated using a decoy-fusion approach in Mascot (version 2.6.0.0). Identified peptide spectrum matches with −10logP value higher than 14 were kept at an FDR threshold of 1%, and proteins identified with less than two peptides were discarded. The mass spectrometry proteomics data have been deposited to the ProteomeXchange Consortium *via* the PRIDE partner repository with the dataset identifier PXD028619.

### 2.4. Biofilm Assays

To compare *A. baumannii* ATCC 17978 and derivatives strains, biofilm formation and metabolic activity were measured using 2,3-bis(2-methoxy-4-nitro-5-sulfophenyl)-5-[(phenylamino)carbonyl]-2H-tetrazolium hydroxide (XTT) assays as previously described (Orsinger-Jacobsen et al., 2013) with some modifications. Briefly, MHB in a 96-well flat-bottomed polystyrene plate was inoculated with 150 μl per well at 5.10^7^ CFU/ml of a fresh overnight culture. The plate was incubated at 37°C without shaking in darkness. After 24 h, the plate was read at 595 nm, and the medium and the pellicle were discarded. Biofilm was washed twice with 200 μl of ultrapure water. XTT solution was added, and samples were incubated for 3 h at 37°C. The optical density (OD) at 490 nm was then measured. Biomass quantification between the different strains was performed by crystal violet (CV) method using a 24-well flat-bottomed polystyrene plate inoculated with 1 ml per well at 5.10^7^ CFU/ml. The plate was incubated at 37°C without shaking in darkness for 48 h. Then, OD at 600 nm of cultures was read. Biofilm was washed once and stained with 1 ml of 0.1% CV for 15 min. After CV removal, wells were washed twice with 1 ml of ultrapure water. CV attached to biomass was solubilized by 1 ml of acetic acid at 30%. Wells were homogenized to measure OD at 580 nm. All assays were performed at least in triplicate in a minimum of three independent experiments.

### 2.5. Confocal Laser Scanning Microscopy

Biofilm formation at the S-L interface was achieved in glass coverslips as described (Le et al., 2021). Briefly, aliquots of 1 ml of bacteria in MHB (inoculum 5.10^7^ CFU/ml) were transferred into each well (24-well flat-bottomed plate) containing a glass coverslip ø12 mm (Supplementary Figure 1). The plate was incubated at 37°C without shaking in darkness for 48 h. The medium was discarded, and biofilms were washed twice with PBS. Biofilms were finally stained with Syto9 (Thermo Fisher Scientific) for 30 min following the manufacturer’s protocol prior to microscopy. Biofilm formation at the solid–liquid–air interface was prepared using a previously described protocol (Fulaz et al., 2019) with some modifications. A 10-ml volume of bacteria in MHB (inoculum 5.10^7^ CFU/ml) was added to a sterile 50-ml Falcon centrifuge tube containing a glass coverslip (24 mm × 50 mm) (Supplementary Figure 1). Biofilm formation in the presence of DNase I from bovine pancreas (Sigma-Aldrich) was performed by supplementing medium with DNase I at 100 μg/ml (Tahrioui et al., 2019). After 24 h of incubation at 37°C without shaking, the coverslip was washed with PBS, and biofilms were stained with Syto9 (Filmtracer LIVE/DEAD Biofilm Viability Kit, Invitrogen). The coverslip was then assembled onto a glass microscope slide using Mowiol 4–88 mounting medium. Image acquisitions were performed using Leica TCS SP8 CFS confocal microscope with fixed stature (Leica Microsystems), equipped with diode laser (Coherent) at 488 nm for Syto9. Fluorescence emission was detected sequentially by a hybrid detector (Leica Microsystems) in photon counting mode with a specific band from 500 to 540 nm for Syto9. Image processing was performed with Imaris software.

### 2.6. Drug Susceptibility Assays

The minimum inhibitory concentrations (MICs) of antibiotics (azithromycin, erythromycin, spiramycin, ticarcillin, erythromycin, colistin, gentamicin, tobramycin, novobiocin, tetracycline, tigecycline, imipenem, and ciprofloxacin; Sigma-Aldrich) and antiseptic (chlorhexidine gluconate, Sigma-Aldrich) on ATCC 17978 WT and derivative strains were determined by the standard microdilution method in MH or MH-cation-adjusted broth using an initial inoculum of 5.10^5^ CFU/ml, as recommended by the Clinical and Laboratory Standards Institute (CLSI) (2015). The minimum biofilm eradication concentration (MBEC), defined as the lowest concentration of an antibiotic that prevents visible growth in the recovery medium used to collect biofilm cells (Macia et al., 2014), was determined using Calgari Biofilm Device (Innovotech, Canada) as previously described (Ceri et al., 1999). Briefly, MH or MH-cation-adjusted broth was inoculated with 10^7^ CFU/ml from an overnight culture in a 96-well plate and incubated at 37°C for 24 h with shaking. Biofilms grew around the plastic pegs on the lid of the plate. Pegs were washed with PBS at 10 mM and challenged with increasing concentrations of antimicrobial agents for an additional 24 h at 37°C. Then, biofilms were washed and removed from pegs by sonication (ultrasonic bath) for 20 min in fresh sterile MHB (recovery plate). The recovery plate was incubated for 24 h at 37°C. OD_650_ of each well was measured to determine MBEC values. MIC and MBEC experiments were performed in three independent assays.

### 2.7. Growth Assays

MHB was inoculated at 10^7^ CFU/ml with fresh overnight cultures of *A. baumannii* ATCC 17978 WT or derivative strains. Strains were grown to mid-log phase and harvested by centrifugation (2,000 × *g* for 5 min). Spotting assay method on supplemented M9 agar plate (Harding et al., 2017) was then used to quantify the impact of 10 mM L-phenylalanine, 256 μg/ml phenylacetic acid (PAA), and 2,048 μg/ml gallic acid (GA) and tannic acid (TA) (Cerqueira et al., 2014; Lin et al., 2015). High-osmolarity adaptation was achieved by measuring for 24 h *A. baumannii* growth in MHB supplemented with 500 mM sucrose (Fluka). Conventional dilution series and plating techniques were carried out to evaluate bacterial survival. Three independent experiments were performed.

### 2.8. Fluorescence Anisotropy Assay

Planktonic or biofilm cultures of *A. baumannii* ATCC 17978 WT, ΔMac, and ΔMac_c were grown in MHB at 37°C for 24 or 48 h, respectively, and cell membrane fluidity was investigated as previously described (Tahrioui et al., 2020). Briefly, cell pellets were washed twice in 10 mM MgSO_4_ and resuspended to reach 0.1 OD_600_. Then, 1 ml of the resuspended cultures was incubated with 4 μM 1,6-diphenyl-1,3,5-hexatriene (DPH; Sigma-Aldrich) in the dark for 30 min at 37°C. Measurement of the fluorescence anisotropy was performed using the Spark 20 M multimode Microplate Reader (Tecan Group Ltd.). Excitation and emission wavelengths were set to 365 and 425 nm, respectively. The anisotropy was calculated according to Lakowicz (2006). The relationship between anisotropy and membrane fluidity is an inverse one, where decreasing anisotropy values correspond to a more fluid lipid membrane and *vice versa*. All values are reported as means of at least triplicate analyses for each experimental variable.

### 2.9. Chrome Azurol S Assay

Quantification of secreted siderophores was performed as previously described (Penwell and Actis, 2019). Briefly, 250 ml Erlenmeyer for preculture and 24-well plate for culture were both conditioned with 0.5 M HCl and then rinsed three times with MiliQ water before sterilization (autoclaving or 30-min UV treatment). For the preculture, 50 ml of the succinate medium (Penwell and Actis, 2019) was inoculated with three colonies and then incubated at 37°C during 48 h under 140 rpm agitation. For the culture, 2 ml of succinate medium were inoculated from the preculture at 0.01 OD_600_ and incubated for 48 h at 37°C without agitation. Then, 1 ml of the culture was centrifuged during 20 min at 10,000 × *g*, and 150 μl of the supernatant were transferred to a 96-well plate, at least in triplicate. Finally, 30 μl of the chrome azurol S (CAS) reagent were added, and kinetic absorbance at 630 nm was performed for 60 min. All assays were repeated three times in triplicate.

### 2.10. Bacterial Adhesion to A549 Human Alveolar Epithelial Cells

A549 human lung adenocarcinoma cells from ATCC were grown as monolayer cultures in Dulbecco’s modified Eagle’s medium (DMEM) or in Ham’s F-12 Nutrient Mixture for at least 20 days to allow differentiation to an alveolar type II (ATII)-like phenotype, as indicated, supplemented with 10% heat-inactivated fetal bovine serum and antibiotics (100 U/ml of penicillin G and 100 μg/ml of streptomycin) (Cooper et al., 2016). Cells were maintained at 37°C in a humidified atmosphere of 5% CO_2_. All cell culture media and supplements were purchased from Thermo Fisher Scientific. Then, cells were trypsinized and transferred to 24-well plates to get a monolayer of 10^5^ cells per well. After 24 h of incubation under the same conditions, A549 cells were washed twice with PBS and fresh medium without antibiotic was added. *A. baumannii* ATCC 17978 WT, ΔMac, and ΔMac_c strains were added to the cells at a ratio of bacteria to host cells of 20:1 [multiplicity of infection (MOI) of 20]. The cells infected with bacteria were incubated at 37°C under an atmosphere of 5% CO_2_ for 5 or 24 h. To determine bacterial adhesion, they were washed five times with PBS, fixed with ice-cold methanol for 20 min, and stained with Giemsa solution. Routinely, 10 microscopic fields were examined along the length of the coverslip. In each field, 10 epithelial cells were examined. The adhesion index was calculated as the total bacterial count divided by 100. The cells were examined using a Nikon Eclipse Ci-S microscope. All assays were repeated three times in triplicate. Student’s *t*-test was performed to evaluate the statistical significance of the observed differences.

### 2.11 Statistical Analysis

Proteomic data were statistically analyzed using Progenesis LC-MS software with ANOVA. Except for the proteomic data, the statistical analyses were carried out with the GraphPad Prism8 software. We used the non-parametric *t*-test, which is a Mann–Whitney test. Mean and standard deviation (SD) calculated from at least three independent experiments were presented.

## 3. Results and Discussion

Biofilms are key microbial ecosystems. To highlight critical determinants of *A. baumannii* sessile lifestyle that can be potential targets against biofilms, we compared protein profiles of bacteria grown in mature S-L biofilms with those of their planktonic stationary phase counterparts. For this comparison, we used the SDF strain, since it does not present classical biofilm determinants (such as Csu pili, PgaABCD, and type IV pili) (Antunes et al., 2011; Eijkelkamp et al., 2014) and may therefore express less characterized and interesting protein systems involved in biofilm development. We compared its proteome with those of the well-studied ATCC 17978 strain that was cultivated in similar conditions.

### 3.1 Proteomic Study of Solid–Liquid Biofilms Formed by SDF and ATCC 17978 Strains

**FIGURE 1:**
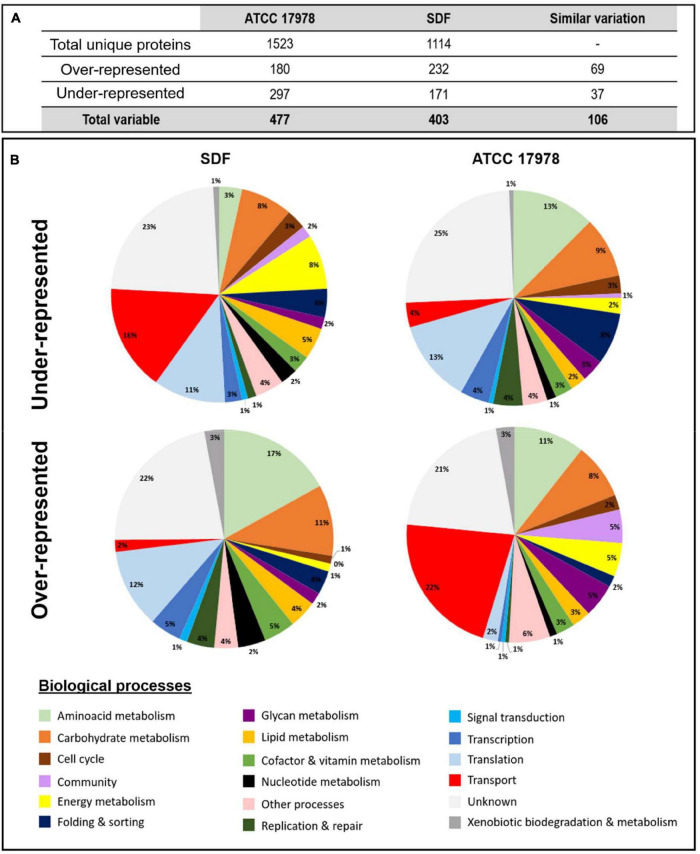
Proteomic analyses of solid–liquid (S-L) biofilms formed by *A. baumannii* ATCC 17978 and SDF strains compared to their planktonic counterparts. **(A)** Number of proteins identified by proteomic analyses of *A. baumannii* ATCC 17978 and SDF strains and number of proteins with modified abundance in S-L biofilms compared to their planktonic counterparts. **(B)** Classification of these differentially represented proteins from *A. baumannii* SDF (left) and ATCC 17978 (right) according to their biological processes using the kyoto encyclopedia of genes and genomes (KEGG) pathway.

The S-L interface is not the favored interface to grow as biofilm for some *A. baumannii* strains (Marti et al., 2011b), and thus, for an optimized biomass–surface ratio, S-L biofilms were grown on glass wool (Crouzet et al., 2017). A slight shaking was performed to prevent the pellicle development that could happen with the ATCC 17978 strain (Kentache et al., 2017). The proteomic quantitative study revealed that among the 1,523 and 1,114 unique proteins identified in the overall samples (planktonic and biofilm) from ATCC 17978 and SDF, respectively (Figure 1A), 477 and 403 proteins showed a significant variation of abundance (fold ≥ 2) according to the mode of growth [Supplementary Table 3 (ATCC) and Supplementary Table 4 (SDF)]. Indeed, two protein populations were distinguished in S-L biofilms: (i) underrepresented and (ii) overrepresented proteins (Figure 1B). Among all the differentially represented proteins, 106 out of 143 proteins common to both analyses presented the same dynamics of variation. Taken together, *A. baumannii* S-L biofilms were characterized by decreased accumulation of proteins involved in bacterial fitness including metabolic proteins and in important surface remodeling such as membrane proteins belonging to transport systems. Indeed, underrepresented proteins were mainly distributed in four functional groups according to the kyoto encyclopedia of genes and genomes (KEGG) pathway: (1) amino acid metabolism (37/297 in ATCC 17978 and 29/171 in SDF), (2) carbohydrate metabolism (27/297 and 18/171, respectively), (3) genetic information processes corresponding to replication and repair, transcription, translation, folding, and sorting (87/297 of underrepresented population in ATCC 17978 and 41/171 in SDF), and (4) unknown functions (74/297 and 38/171 proteins, respectively). Even though in both strains overrepresented proteins were more heterogeneously distributed (Figure 1B), we were able to distinguish two main groups: (1) proteins involved in transport systems (39/180 in ATCC 17978 and 36/232 in SDF) and (2) proteins with unknown functions (37/180 of overrepresented proteins in ATCC 17978 and 54/232 overrepresented proteins in SDF).

### 3.2 Specific Determinants of SDF Solid-Liquid Biofilm

We looked for specific determinants of SDF S-L biofilm to understand how this strain could produce and maintain a biofilm as dense as the reference strain ATCC 17978, while lacking the main critical biofilm determinants. Of interest, SDF S-L biofilm was characterized by the accumulation of proteins involved in translation (Figure 1B). Indeed, the SDF strain accumulated 22 ribosomal subunits in S-L biofilm (with maximum fold changes ranging from 3.3 to 48; Supplementary Table 4). These proteins are usually characteristics of the physiological growing state (Bosdriesz et al., 2015) like in ATCC 17978 planktonic growth mode (Cabral et al., 2011; Kentache et al., 2017). However, analysis of *P. aeruginosa* PAO1 biofilms showed that the ribosomic mRNA expression level was stably maintained between dividing and slowly growing cells (Williamson et al., 2012). The importance of these ribosomal proteins was also reported in *Bacillus subtilis* where the deletion of *rpsU* and *rpsK* genes highly decreased biofilm formation (Takada et al., 2014). Even though S-L biofilms were 4 days old, we cannot exclude that the SDF strain continues to divide, with a substantial part of ribosomes translating community determinants.

In addition, our analysis also revealed the accumulation of 20 proteins being part of the energy metabolism and that belong to the complex I (NuoABCDFGHI, 7.7- to 37-fold changes), the complex II (SdhCAB, 17- to 252-fold changes), the cytochrome b0 oxidase (CyoAB, 9- and 57-fold changes, respectively) or the F1F0 ATP synthase (3 proteins) and three ubiquinone biosynthesis proteins (UbiG, 10-fold; UbiB, 18-fold; and UbiE, fivefold), and the flavoprotein-ubiquinone oxidoreductase EftD (13-fold), indicating that the function of the energetic respiratory chain was strongly exacerbated here. This suggests that a high ATP requirement and a potential redox regulation may increase the membrane potential to promote biofilm development (Qin et al., 2019).

Finally, we observed the overexpression of 11 proteins involved in the lipid metabolism, several of them being enzymes implicated in complex lipid biosyntheses. For example, PlsB (13-fold change) synthesizes phosphatidic acid precursors (Lehner and Kuksis, 1996), which are essential for the adaptation to environmental stresses through the modification of membrane composition and fluidity (Dubois-Brissonnet et al., 2016; Tao et al., 2021). As already reported, it may also contribute to modify the SDF adhesiveness character (Benamara et al., 2011; Lattif et al., 2011; Dubois-Brissonnet et al., 2016). Other enzymes, like Acr1 (54-fold change) and Wax-dgaT (fivefold change) are characteristics of environmental bacteria. The bifunctional acyltransferase Wax-dgaT is involved in the synthesis of triacylglycerols (TAGs) from diacylglycerols as well as in the synthesis of wax-ester (WE) (Lehner and Kuksis, 1996; Ishige et al., 2002). Accumulation of Wax-dgaT might so promote lipid storage as a carbon source to survive in nutrient deprivation. TAG storage could also contribute to bacterial desiccation tolerance (Alvarez et al., 2004; Alvarez, 2016).

This S-L biofilm proteomic quantitative analysis showed that SDF did not synthesize adhesion/community determinants known to be expressed in *A. baumannii*, like the Acinetin locus, the Csu pili or the P pilus or the PNAG polymer transporter PgaA, determinants detected in ATCC 17978 S-L biofilm. Interestingly, two systems may participate to the maintenance and the cohesion of the SDF S-L biofilm: (i) the type III *pilus* adhesion factors, FilC (71-fold change) and FilF (12-fold change), already described in ATCC 17978 pellicle communities (Marti et al., 2011b; Kentache et al., 2017), and (ii) a two-partner secretion system (TPS) FhaB/C. Indeed, we identified the protein ABSDF3544 (fourfold change), which is a hemagglutinin/hemolysin type protein. It may correspond to the secreted protein of a TPS system, with FhaB being the passenger domain and FhaC, the translocator (identified here with a 17.8-fold change). ABSDF3544 is not conserved between *A. baumannii* species, but it had two homologous in the SDF genome (not identified here). In *A. baumannii*, FhaB/C systems are involved in the adhesion to human epithelial and bronchial cells (Astaneh et al., 2014, 2017; Pérez et al., 2017). In AbH12O-A2 strain, the exoprotein AbFhaB (also called TpsA, 31.8% id. to ABSDF3544) contributes to the tridimensional *A. baumannii* aggregation (Pérez et al., 2017). Here, ABSDF3544 may participate in cell–cell interactions but also in SDF biofilm formation, as it was already shown for FhaB/C system in *Bordetella pertussis* (Serra et al., 2011).

### 3.3 Common Determinants of Solid–Liquid Biofilm Formation

Despite the small genome of SDF (3.2 Mb and 3,050 open reading frames) (Fournier et al., 2006), we highlighted 69 commonly overexpressed proteins in both ATCC 17978 and SDF S-L biofilm cells. Among them, we identified proteins belonging to already characterized metabolic pathways and several systems necessary for environmental exchanges in sessile bacteria such as: (*i)* arginine catabolism (Cabral et al., 2011; Kentache et al., 2017), (*ii)* some adhesion factors like OmpA (Gaddy et al., 2009), (*iii)* the polysaccharide export system (Wza-Wzc-Wzi) (Kenyon and Hall, 2013), (*iv*) the T6SS secretion system, and (*v)* transport systems for surface modulation (Bam, Tam) or environmental exchanges (OmpW, Omp25, OprD, CarO, and ABYAL0223 porins) and also the AdeIJK efflux pump (Kentache et al., 2017). Interestingly our comparative analysis revealed that both SDF and ATCC 17978 S-L biofilm cells overexpressed also two proteins of a tripartite efflux pump ABSDF2985 and ABSDF2983 (40- and 22-fold changes, respectively) and ABYAL0573-74 and ABYAL0571 (four and sixfold changes, respectively) that were annotated MacAB-TolC. The overexpression of the *A1S_0538* gene from this system was also highlighted in a transcriptomic approach of *A. baumannii* ATCC1978 24-h S-L biofilms (Rumbo-Feal et al., 2013).

In *A. baumannii*, the MacAB-TolC system is a tripartite efflux pump where MacB is an atypical ABC family transporter with a recently determined atomic structure (Okada et al., 2017), MacA is a membrane fusion protein, and TolC is an outer membrane protein. This well-conserved system was first identified in *E. coli* (Kobayashi et al., 2001). It handles the efflux of substrates either from the periplasm and/or from the cytoplasm to the extracellular environment of the bacterial cell (Crow et al., 2017; Fitzpatrick et al., 2017). For many species like *E. coli*, *Stenotrophomonas maltophilia*, or *K. pneumoniae*, it is involved in the resistance to macrolides, aminoglycosides, polymyxins, and cyclines (Lin Y. T. et al., 2014; Fitzpatrick et al., 2017; Zheng et al., 2018). In addition, MacAB was shown to extrude various compounds such as toxins (enterotoxin STII in *E*. *coli*; Yamanaka et al., 2008), protoporphyrin IX (Turlin et al., 2014), or lipopeptides and siderophores in *Pseudomonas* species (Imperi et al., 2009; Greene et al., 2018) and can be involved in virulence (Nishino et al., 2006). MacAB was recently described to protect *Salmonella enterica* serovar typhimurium from oxidative stress through linearized siderophore product secretion (Bogomolnaya et al., 2020). Thus, the MacAB system appears to fulfill numerous transport functions for a wide range of substrates. In addition, its contribution to biofilm formation has been described for *S. maltophilia* (Lin Y. T. et al., 2014). In *A. baumannii*, MacAB-TolC shares 83% of amino acid sequence similarity with its *E. coli* counterpart and, therefore, may be involved in the efflux of macrolides and novobiocin (Okada et al., 2017; Pérez-Varela et al., 2019). So far, to our knowledge, the function of the MacAB-TolC pump in *A. baumannii* biofilm cells has never been considered.

### 3.4 Involvement of MacAB-TolC in Biofilm Formation

To unravel the function of MacAB-TolC tripartite efflux system of *A. baumannii* in biofilm formation, we have attempted unsuccessfully to generate a Δ*macAB-tolC* deletion mutant in the SDF strain. We, however, succeeded to make this deletion mutant (ΔMac) in the ATCC 17978 strain and also generated a complemented strain (ΔMac_c) harboring pWH1266::*macAB-tolC*. We checked that deletion of *macAB-tolC* did not affect growth of ΔMac mutant neither in planktonic nor in biofilm cultures (Supplementary Figure 2). For ΔMac_c strain, the cell metabolic activity was slightly decreased in biofilm probably due to the presence of the complementation plasmid, since the same phenotype was observed in the strain carrying the empty plasmid (Supplementary Figure 2).

**FIGURE 2:**
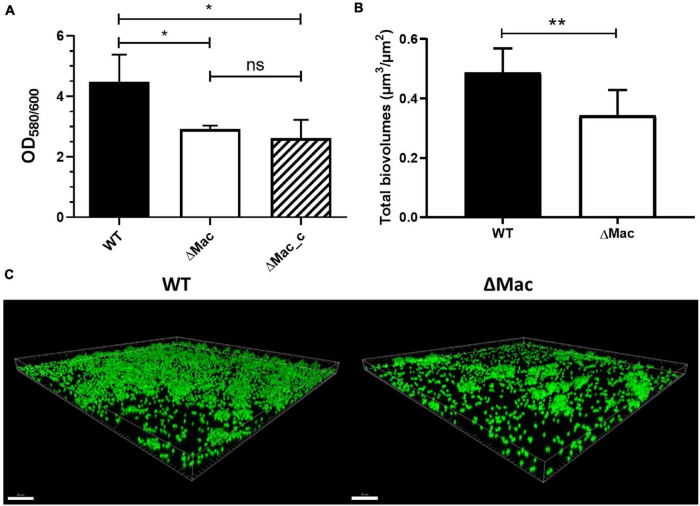
Impact of the *macAB-tolC* deletion on *A. baumannii* biofilm formation at the solid–liquid interface. **(A)** Biomass quantification by crystal violet staining of *A. baumannii* ATCC 17978 wild type (WT), ΔMac, and ΔMac_c (ΔMac complemented strain). **(B)** Quantification of biovolume (μm^3^/μm^2^) of *A. baumannii* ATCC 17978 WT and ΔMac biofilms based on the confocal fluorescence images. **(C)** Representative confocal laser scanning microscopy (CLSM) images of *A. baumannii* ATCC 17978 WT (left) and ΔMac biofilms (right) labeled with Syto9. Data shown represent mean values (±SD) from at least three independent biological experiments (ns, not significant; *p < 0.05; **p < 0.01). Scale bar: 20 μm. OD, optical density.

Interestingly, our results showed that deletion of the Mac system negatively affected the biomass amount that was decreased by 33% (*p* < 0.05) after 48 h of biofilm growth (Figure 2A). Complementation did not, however, restore the phenotype to a level comparable to the level reached by the WT strain. Since the antibiotic selection pressure was not applied in our experiment owing to its influence on biofilm formation (Peleg et al., 2008; Penesyan et al., 2019), the complementation plasmid may have been lost within the time frame of the experiment. We, however, failed to demonstrate by numeration a difference between the ΔMac and the ΔMac_c strains (data not shown). In parallel, confocal laser scanning microscopy (CLSM) analysis of biofilms was performed using Syto9 staining. Again, the total biovolume subsequent to the ΔMac deletion was reduced by 23% after 48 h biofilm formation (Figures 2B,C). Our results are consistent with the study performed in *S. maltophilia*, where the deletion of the MacAB-TolC system induces a 48% decrease of biofilm formation (Lin Y. T. et al., 2014). Taken together, inactivation of genes encoding the MacAB-TolC tripartite efflux system of *A. baumannii* results in impaired biofilm formation.

### 3.5 Involvement of MacAB-TolC in Antibiotic Resistance

**FIGURE 3:**
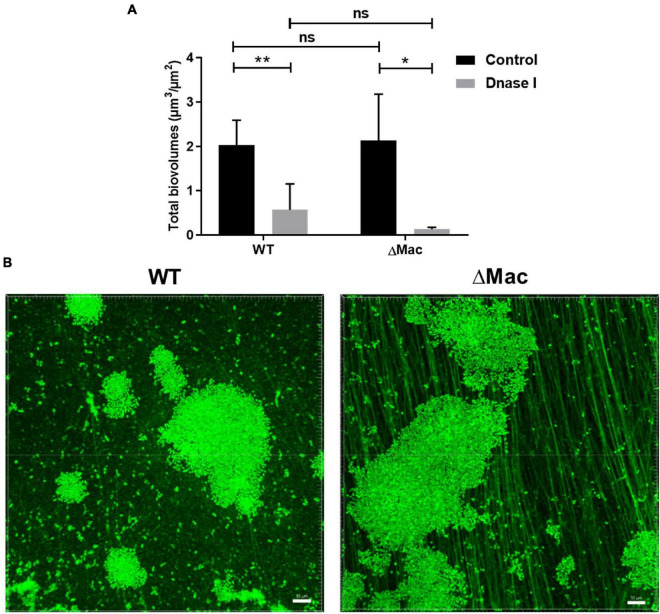
Impact of *macAB-tolC* deletion on biofilm formation at the solid-liquid-air interface. **(A)** Total biovolume (μm^3^/μm^2^) of 24-h-old *A. baumannii* ATCC 17978 WT and ΔMac biofilms after exposure to DNase I (100 μg/ml) compared to untreated control biofilms. Quantification of biovolume was based on the confocal fluorescence images of Syto9 stained biofilms. Data shown represent mean values (±SD) from at least three independent biological experiments (ns, not significant; *p < 0.05; **p < 0.01). **(B)** Representative confocal fluorescence images of *A. baumannii* ATCC 17978 WT (left) and ΔMac biofilms (right) labeled with Syto9 at the solid–liquid–air interface (three-phase interface). Image construction was carried out using Imaris software. Scale bar: 10 μm.

As the MacAB-TolC system was already shown to contribute to the antibiotic resistance in *A. baumannii* and various bacterial species (Greene et al., 2018; Pérez-Varela et al., 2019), we compared MICs of WT and ΔMac strains. In the present study, we did not observe any difference between both strains (Supplementary Table 5). The deletion of the MacB transporter was, however, shown to be associated with a slight decrease in erythromycin (from 4 to 2 μg/ml) and novobiocin MICs (from 8 to 2 μg/ml; Pérez-Varela et al., 2019). This discrepancy might be linked to the deletion of the entire system instead of *macB* alone. Here, MacAB-TolC does not seem to participate in the antibiotic efflux in *A. baumannii* in a planktonic growth mode or the expression of another efflux pump may counteract the deletion of the Mac system in the ΔMac strain. Regarding the antibiotic tolerance in biofilms, MBEC assays revealed that ΔMac was surprisingly more tolerant to aminoglycosides, such as gentamicin (128 μg/ml) and tobramycin (64 μg/ml), than the WT strain (32 μg/ml). ΔMac_c strain showed a restored phenotype with MBEC at 16 μg/ml for gentamicin and 32 μg/ml for tobramycin. Hence, to investigate this difference, a biofilm model at the solid–liquid–air interface (three-phase interface), similar to the one present in the Calgary biofilm device, was performed for CLSM imaging (Supplementary Figure 1). After 24 h of incubation, all bacteria (live and dead cells) and biofilm matrices were stained with Syto9. There was no difference in the biovolume at the three-phase interface (Figure 3A) contrary to the one observed at the S-L interface (Figure 2B). This is consistent with our proteomic data pointing out that the overexpression of MacAB-TolC happens essentially when *A. baumannii* grows at the S-L interface and not in pellicle (Kentache et al., 2017). However, ΔMac biofilm images at the three-phase interface showed a well-developed fiber-like network within the EPS matrix. These fibers are extracellular DNA (eDNA), since this component was labeled with Syto9 and was also degraded by DNAse I (Figure 3). Indeed, DNase I treatment led to a significant reduction of biofilm formation for both WT and ΔMac (72 and 95%, respectively) after 24-h incubation (Figure 3A). In *A. baumannii*, eDNA was shown to be released either by an active mode, in a free form or encapsulated in membrane vesicles during early biofilm growth phase, or by cell lysis contributing to the regrowth of freshly dispersed cells (Sahu et al., 2012). A kinetic profile of biofilm formation at the three-phase interface was also performed in our study. As these eDNA-containing fibers were mainly observed from 24 h of growth (data not shown), these eDNA fibers may mainly originate from cell lysis.

It has been largely reported that aminoglycosides could have an impaired penetration in *P. aeruginosa* biofilms due to their interaction with negative components of the matrix (alginate or eDNA; Alipour et al., 2009). In line with this observation, exogenous DNA addition to biofilm growth medium was shown to provide a shield effect against aminoglycosides (Chiang et al., 2013). Here, in the ΔMac mutant, it is likely that an excess of eDNA could promote divalent cation sequestration and block aminoglycoside antibiotic diffusion within the matrix, thus explaining the observed tobramycin and gentamicin tolerance increase (Tseng et al., 2013). Although colistin can interfere with the electrostatic network of the EPS matrix (Klinger-Strobel et al., 2017), eDNA does not seem here to induce a colistin biofilm tolerance.

### 3.6 MacAB-TolC Contributes to the Envelope Stress Response

Gram-negative bacteria possess a complex envelope to adapt their physiology to environmental conditions. This adaptation is highly controlled by two-component systems (TCSs) (De Silva and Kumar, 2019). In *A. baumannii*, MacAB-TolC is regulated by BaeSR, a TCS that detects environmental stresses, like specific envelope-damaging agents and high osmolarity conditions. Moreover, BaeSR modulates the expression of other efflux pumps such as AdeIJK and AdeABC, which are involved in cell detoxification and maintenance (Lin M. F. et al., 2014; Lin et al., 2015).

Lin et al. (2015) have demonstrated by phenotype microarray experiment the Δ*baeR* mutant susceptibility and an upregulation of *macB* (6.2-fold) in response to a tannic acid (TA) treatment. Herein, we determined TA MICs on WT and ΔMac deletion mutant and found accordingly that the WT strain was more resistant to TA (512 μg/ml) than the ΔMac strain (128 μg/ml). Complementation partially restored the resistance to this tannin (256 μg/ml in ΔMac_c). The ΔMac susceptibility to TA was confirmed using spot assay (Supplementary Figure 3). At least 10^8^ CFU/ml of ΔMac were inhibited by 2,048 μg/ml of TA, whereas the same concentration of tannin inhibited only 10^5^ CFU/ml of WT and 10^6^ CFU/ml of the complemented strain. The same experiments were performed with GA, a degradation product of TA (Tahmourespour et al., 2016), but no significant difference between the WT and the ΔMac strains was observed (Supplementary Figure 3). Thus, MacAB-TolC may be required to allow *A. baumannii* survival in the presence of high doses of TA through either a TA efflux or a degradation process. Since TA is also an iron-chelating and antioxidant agent (Lin et al., 2015, 2020), the incapacity of the ΔMac strain to survive at high doses of TA could also be due to its impairment in maintaining iron homeostasis.

**FIGURE 4:**
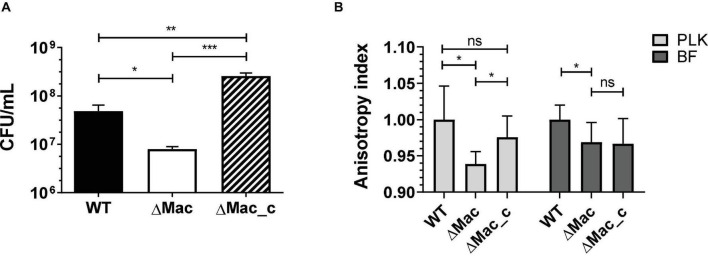
Tolerance to sucrose and membrane fluidity analyses of WT vs. ΔMac and ΔMac_c derivative strains of *A. baumannii*. **(A)** The 24 h growth measurements in high-osmolarity condition (500 mM sucrose). **(B)** Fluorescence anisotropy measurements of planktonic (PLK) growth culture (24 h; clear gray) or biofilm (BF) growth culture (48 h; dark gray). Data shown represent mean values (±SD) from at least three independent experiments. The data were statistically analyzed using unpaired *t*-test to calculate *p*-values (ns, not significant; *p < 0.05; **p < 0.01; ***p < 0.001). CFU/mL, colony-forming unit per ml.

Moreover, we compared the tolerance to envelope stress of the WT, ΔMac, and ΔMac_c strains in high-osmolarity conditions. Interestingly, the ΔMac mutant was less tolerant to 500 mM sucrose after 24 h of growth as compared to the WT strain (Figure 4A). Susceptibility to this high-osmolarity condition was restored in ΔMac_c complemented mutant. These results are consistent with the study demonstrating that the expression of *baeR* was increased by twofold in response to 20% sucrose (Lin M. F. et al., 2014). To counteract high-osmolarity conditions and to survive this environmental stress, microorganisms may change their membrane composition, thus impacting membrane fluidity (Beney and Gervais, 2001). Accordingly, we measured the membrane fluidity of WT, ΔMac, and ΔMac_c strains by fluorescence anisotropy method (Figure 4B). The anisotropy index indicated that the ΔMac mutant presented a higher membrane fluidity than that in the WT strain, either in planktonic suspension or in biofilm. The membrane fluidity of ΔMac_c was partially and significantly restored as compared to the WT one, in planktonic but not in sessile lifestyle, probably due to the lack of antibiotic selection pressure.

These results demonstrated that MacAB-TolC contributes to maintain WT membrane rigidity and allow the tolerance to high-osmolarity conditions. This is in agreement with the study of Henry et al. (2012), highlighting that the rebuilding and rigidity maintenance of the membrane of a lipopolysaccharide (LPS)-deficient mutant is concomitant with the overexpression (28- to 39-fold) of the *macAB-tolC* system under the regulation of BaeS/R. In a similar manner, colistin treatment causing major membrane damages, i.e., an important envelope stress, induced the overexpression of *macAB-tolC* as a cell wall maintenance response (Henry et al., 2014). Of note, the *emrAB* efflux pump contributes in a similar manner to osmotic stress and colistin resistance in *A. baumannii* (Lin et al., 2017). Recently, it was also reported to be involved in biofilm formation (Lin et al., 2020). As mentioned above, BaeS/R positively regulates AdeIJK and AdeABC together with MacAB-TolC (Lin M. F. et al., 2014; Lin et al., 2015). In our proteomic data (Supplementary Tables 3, 4), these efflux pumps were overrepresented in biofilm and could therefore contribute to antibiotic tolerance in this mode of growth.

When the deletion of AdeB led to a significant decrease of biofilm formation similarly to what the deletion of MacAB-TolC did, the deletion of BaeR moderately impacted biofilm development (Lin et al., 2020). It is tempting to speculate that an effective antibiofilm strategy would be directed toward the design of a broad efflux pump inhibitor rather than to prevent a TCS activity.

### 3.7 Biofilm Proteome Reveals Disrupted Iron Homeostasis in ΔMac

To further understand the contribution of MacAB-TolC to *A. baumannii* biofilm formation, we proceeded to a large-scale proteomic analysis of WT and ΔMac strains in the conditions used to highlight MacAB-TolC overexpression (see *Proteomic Analyses of Planktonic and Sessile Bacterial Cultures* section). We analyzed intracellular and membrane compartments and identified 48 proteins with varying expression levels. Among them, 37 proteins were underexpressed (Table 1), whereas 11 were overexpressed in the ΔMac strain (Table 2).

**TABLE 1:** Proteins under-represented in solid–liquid biofilm of ΔMac.

Label ABYAL	Label A1S_	Fold change	Gene	Description	Fraction	Peptides	Confidence	Anova (*p*)
				**MacAB-TolC system**				
ABYAL0571	A1S_0535	18.0	*tolC*	Outer membrane protein	M	2	89.64	1.1E-03
ABYAL0574	A1S_0538	12.9	*macA*	ABC tripartite efflux pump membrane fusion protein	M	5	216.38	1.5E-10
				**Siderophore**				
ABYAL1976	A1S_1657	2.6	*bfnL*	Baumannoferrin biosynthesis protein	I	3	137.34	5.1E-03
ABYAL2846	A1S_2375	3.1	*barB*	Siderophore ABC transporter	M	13	716.65	1.6E-03
ABYAL2852	A1S_2380	3.2	*basF*	Isochorismatase	I+M	3	109.43	3.8E-03
ABYAL2853	A1S_2381	2.8	*basE*	2,3-Dihydroxybenzoate-AMP ligase/S-dihydroxybenzoyltransferase	I	2	168.79	5.1E-03
ABYAL2858	A1S_2386	2.3	*bauB*	Acinetobactin periplasmic binding protein	M	7	389.76	7.5E-03
ABYAL2863	A1S_2390	2.3	*basB*	Acinetobactin biosynthesis protein	M	3	161.11	7.6E-03
				**Sulfur**				
ABYAL0038	A1S_0028	2.2	*ssuD*	FMNH_(2)_-dependent alkanesulfonate monooxygenase	I+M	4	202.00	3.4E-03
ABYAL0039	A1S_0029	2.5	*ssuA*	Aliphatic sulfonate ABC transporter periplasmic	M	2	103.65	4.2E-04
ABYAL0040	A1S_0030	2.5	*ssuA*	Aliphatic sulfonate ABC transporter periplasmic	M	2	88.15	1.3E-03
ABYAL3025	A1S_2537	2.3	*ssuR*	DNA-binding transcriptional activator (LysR-family)	M	3	147.98	2.1E-04
ABYAL3888	A1S_3305	2.1	*msuE*	NADH-dependent FMN reductase	M	2	96.85	3.7E-03
ABYAL3889	A1S_3306	3.1	*msuD*	FMNH_(2)_-dependent dimethylsulfone monooxygenase	I+M	4	259.99	3.8E-04
ABYAL1751	A1S_1485	2.4	*metQ*	Methionine ABC transporter permease	M	8	392.49	4.3E-04
				**PAA degradation**				
ABYAL1576	A1S_1335	15.2	*paaZ*	Oxepin-CoA hydrolase/dehydrosuberyl-CoA semialdehyde dehydrogenase	I+M	5	221.52	4.9E-03
ABYAL1577	A1S_1336	9.6	*paaA*	1,2-phenylacetyl-CoA epoxidase subunit A	I	3	113.1	7.7E-04
ABYAL1583	A1S_1342	9.2	*paaG*	2-(1,2-epoxy-1.2-dihydrophenyl)acetyl-CoA isomerase	I+M	8	394.61	3.1E-03
ABYAL1584	A1S_1343	9.6	*paaH*	3-hydroxybutyryl-CoA dehydrogenase	I	3	178.95	2.6E-03
ABYAL1585	A1S_1344	12.4	*paaJ*	Beta-ketoadipyl-CoA thiolase	I	2	123.36	6.4E-03
				**Secretion system**				
ABYAL1534	A1S_1296	2.5	*hcp1*	Type VI secretion system effector	M	14	1017.28	2.6E-07
ABYAL3106	A1S_2602	4.1	*rbtA*	Rhombotarget A	M	3	102.26	3.2E-04
				**Others**				
ABYAL0608	A1S_0569	2.0		Short-chain dehydrogenase/reductase	M	2	86.33	1.1E-03
ABYAL1300	A1S_1126	3.6		Baeyer–Villiger monooxygenase	M	3	149.25	1.5E-04
ABYAL1493	A1S_1264	2.2		Class A β-lactamase-related serine hydrolase	M	2	105.9	1.4E-03
ABYAL1530	A1S_1292	2.4		Conserved hypothetical protein	M	2	48.99	3.4E-05
ABYAL1698	A1S_1439	2.3		Luciferase-like monooxygenase	I	2	129.35	9.8E-05
ABYAL1742	A1S_1478	2.4		Conserved hypothetical protein	I	3	128.33	9.3E-05
ABYAL1831	A1S_1551	2.1	*parA*	ATPase chromosome partitioning protein	M	2	127.04	8.0E-05
ABYAL2029	A1S_1700	2.0	*acoB*	Acetoin:2,6-dichlorophenolindophenol oxidoreductase beta subunit	M	2	127.54	1.5E-03
ABYAL2289	A1S_1922	2.5		Ribokinase	M	2	96.2	3.3E-04
ABYAL2361		2.2		Conserved hypothetical protein	I	3	127.91	2.2E-03
ABYAL2518	A1S_2084	3.4	*pheA*	Secreted chorismate mutase	M	3	180.53	1.8E-05
ABYAL2931	A1S_2452	3.6	*styD*	Phenylacetaldehyde dehydrogenase	I+M	8	448.54	1.3E-04
ABYAL3342	A1S_2820	2.8		Conserved hypothetical protein	I	2	122.24	1.7E-03
ABYAL3515	A1S_2957	2.2		Zn-dependent hydrolase	M	2	101.57	2.3E-05
ABYAL4020	A1S_3418	2.7	*hpd*	4-hydroxyphenylpyruvate dioxygenase	I	4	210.88	6.0E-03

*“M” for membrane fraction and “I” for intracellular fraction.*

**TABLE 2:** Over-represented proteins in solid–liquid biofilm of ΔMac.

Label ABYAL	Label A1S_	Fold change	Gene	Description	Fraction	Peptides	Confidence	Anova (*p*)
				**Quorum sensing**				
ABYAL0138	A1S_0115	2.7		Non-ribosomal peptide synthetase (NRPS)	I	2	93.95	9.4E-04
ABYAL0139	A1S_0116	2.3		Resistance-nodulation-division (RND) transporter (Ac-505 secretion)	M	5	259.28	2.0E-03
				**Others**				
ABYAL1401	A1S_1191	2.1	*pyrX*	Aspartate carbamoyltransferase	M	4	214.67	1.6E-03
ABYAL1402	A1S_1192	2.3	*pyrX*	Aspartate carbamoyltransferase	M	2	169.65	4.2E-05
ABYAL1640	A1S_1387	2.0	*yhdF*	Short-chain dehydrogenase reductase	M	2	56.02	9.3E-03
ABYAL2806	A1S_2338	2.2	*maeB*	Malate dehydrogenase	M	20	1084.58	2.4E-05
ABYAL2984	A1S_2501	2.2	*gap*	Glyceraldehyde-3-phosphate dehydrogenase	I	2	97.82	1.3E-05
ABYAL3089	A1S_2586	2.0	*dgt2*	Deoxyguanosinetriphosphate triphosphohydrolase-like protein	M	2	92.13	6.3E-04
ABYAL3806	A1S_3231	2.7	*cat*	Succinyl-CoA coenzyme A transferase	I	2	97.74	4.1E-06
ABYAL3914	A1S_3327	2.2	*aceF*	Dihydrolipoamide acyltransferase (E2) component	M	10	587.81	2.8E-05
ABYAL4005	A1S_3403	2.0	*hutI*	Imidazolonepropionase	I	3	147.31	1.0E-04

*“M” for membrane fraction and “I” for intracellular fraction.*

All bacterial cells need iron and have thus developed iron-uptake pathways to scavenge iron from their host during infection. *A. baumannii* ATCC 17978 produces up to 10 siderophores that chelate iron with high affinity from three different loci, named acinetobactin, baumannoferrin (A and B), and the fimsbactins (A–F) (Sheldon and Skaar, 2020). Acinetobactin and the fimsbactins are mixed catechol-hydroxamate-type siderophores, whereas baumannoferrin has solely a hydroxamate-type structure (Yamamoto et al., 1994; Proschak et al., 2013; Penwell et al., 2015). It was already reported that these siderophores and iron requirement were critical for the development of *A. baumannii* communities (Nait Chabane et al., 2014; Kentache et al., 2017). Accordingly, the ATCC 17978 S-L biofilm proteomic analysis pointed out the underexpression of the negative regulator Fur (Ferric uptake regulator). It inhibits the expression of siderophore synthesis and promoted iron storage (Cornelis et al., 2011). Consistently, BauA (10-fold) was involved in acinetobactin import, and proteins involved in baumannoferrin transport (BfnH, threefold; TonB-dependent receptor, 26-fold) were accumulated (Supplementary Table 3).

Interestingly, in the ΔMac proteome analysis, the BfnL protein, involved in the biosynthesis of the baumannoferrin, and five other proteins related to the acinetobactin locus (BasB, BasE, and BasF for biosynthesis; BauB for import; and BarB for export) were underexpressed compared to those in the WT strain (Table 1). However, a potential decrease of baumannoferrin biosynthesis in ΔMac should be considered with caution, since only the BfnL amount decreased (Sheldon and Skaar, 2020). Nevertheless, there was no doubt regarding the decrease of acinetobactin production. Likewise, in *P. aeruginosa*, a deletion mutant of PvdRT-OpmQ, an efflux pump sharing a high structural similarity with MacAB-TolC, presented a downregulation of pyoverdin biosynthesis (Imperi et al., 2009). This PvdRT-OpmQ system was proposed to be responsible for pyoverdin recycling and/or required for its secretion when newly synthesized (Imperi et al., 2009; Hannauer et al., 2010). It is thus tempting to propose that the MacAB-TolC system could participate in the secretion and/or recycling of acinetobactin from the periplasm to the extracellular medium. We performed CAS assays to examine this hypothesis, but as already mentioned (Sheldon and Skaar, 2020), we did not detect any variation of siderophore activity in ΔMac compared to WT in disrupting only the acinetobactin pathway (Supplementary Figure 4A).

In ΔMac, the sulfonate-sulfur utilization step of cysteine biosynthesis pathway was notably affected with the downregulation of five proteins (Table 1) with: (*i*) *and* (*ii*) SsuA involved in aliphatic sulfonate import and SsuD involved in desulfonation of aliphatic sulfonates, both are members of the SsuEADCB system; (*iii*) SsuR the regulator of this system; (*iv*) *and* (*v*) MsuE and MsuD also involved in desulfonation of other aliphatic sulfonate (Kertesz et al., 1999). Moreover, MetQ responsible for the methionine import, an organosulfur source other than sulfonates (Kertesz et al., 1999), was also underexpressed (Table 1). Altogether, these results showed a potential decrease of the cysteine biosynthesis. This particular amino acid is crucial for [Fe-S] cluster biosynthesis. Indeed, iron–sulfur cluster (ISC) and sulfur formation (SUF) pathways, directly regulated by cellular iron status, use free cysteine to liberate sulfur atoms for [Fe-S] cluster assembly (Guédon and Martin-Verstraete, 2006; Ayala-Castro et al., 2008). In ΔMac, iron homeostasis deregulation might compromise [Fe-S] cluster status. Of interest, IscR regulator of [Fe-S] cluster synthesis (Ayala-Castro et al., 2008) and two chaperones involved in this pathway, HscA (3-fold) and HscB (4-fold) were downregulated in ATCC 17978 S-L biofilm (Supplementary Table 3) consistently with an iron limitation in these growth conditions (Vickery and Cupp-Vickery, 2007).

Finally, the highest protein fold changes (up to 15-fold) in ΔMac were obtained for five proteins: PaaA, PaaG, PaaZ, PaaJ, and PaaH, which were highly underrepresented (Table 1). These proteins belong to the phenylacetate (PAA) catabolic pathway that allows the degradation of aromatic compounds to produce acetyl-coA and succinyl-coA for the trichloroacetic acid (TCA) cycle (Teufel et al., 2010; Cerqueira et al., 2014). Considering the function of this pathway, we investigated the capacity of ΔMac to grow on M9 agar plate with phenylacetic acid or phenylalanine. We did not observe a difference between the ΔMac and the WT strains (Supplementary Figure 4B). However, iron availability and/or the unbalanced cellular iron status of the ΔMac mutant may influence the synthesis of this operon as described (Nwugo et al., 2011). Here, the unbalanced cellular iron status on the ΔMac mutant may also influence the PAA expression. It is known that the PAA catabolic pathway, under the control of the global virulence regulator GacA, is involved in *A. baumannii* virulence (Cerqueira et al., 2014). Virulence assays conducted on the ΔMac strain in the model organism *Caenorhabditis elegans* model did not, however, allow us to observe attenuated virulence compared to WT (data not shown, Pérez-Varela et al., 2019). Similarly, in the ATCC 17978 S-L biofilm, five proteins of the PAA pathway and GacA were downregulated (Supplementary Table 3). This is remarkably different from our proteomic analysis of *A. baumannii* pellicle (Kentache et al., 2017) and suggests that *A. baumannii* virulence mediated by *paa* locus and GacA is strictly associated with pellicle formation (Marti et al., 2011a).

### 3.8 MacAB-TolC Limits Adhesion to Human Alveolar Epithelial Cells

**FIGURE 5:**
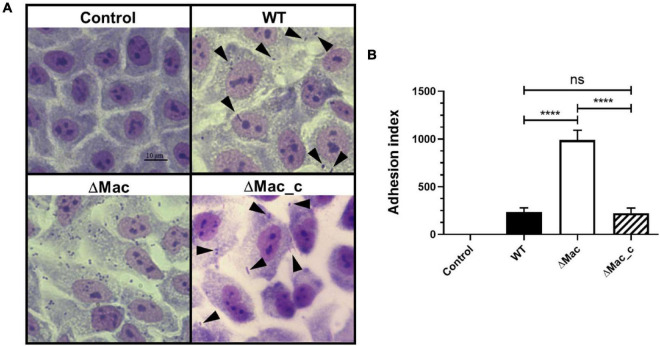
Adhesion of *A. baumannii* to A549 human alveolar epithelial cells. **(A)** ATII-like phenotype A549 differentiated cells were infected with *A. baumannii* ATCC 17978 (WT), ΔMac, and ΔMac_c (ΔMac complemented) at a multiplicity of infection (MOI) of 20. Negative control corresponds to cells with no bacteria added. Black arrows indicate bacteria attached to A549 cells (magnification, ×400). **(B)** Quantification of the adherence of *A. baumannii* ATCC 17978 WT, ΔMac, and ΔMac_c strains to A549 cells. Index of attached bacteria after 24 h of infection. Data shown represent mean values (±SD) from at least three independent experiments performed at least in triplicate. Student’s *t*-test was used to validate the experimental data (ns, not significant; ****p < 0.0001).

Bacterial adherence to target cells is the first step of the infectious process. Interestingly, in our proteomic study, we identified two proteins, ABYAL0138 (A1S_0115, 2.7-fold) and ABYAL0139 (A1S_0116, 2.3-fold), overrepresented in the ΔMac mutant that are involved in the synthesis and transport of acinetin. They are part of the *A1S_0112-A1S_0119* operon already described to contribute to biofilm formation and potential acinetin secretion but also to the interaction with eukaryotic cells and in virulence (Rumbo-Feal et al., 2017). The biological effect of the *macAB-tolC* deletion was therefore tested using A549 human alveolar epithelial cells as a model, since they represent a host cell that could be targeted by *A. baumannii* during respiratory infections. The ΔMac strain shows a remarkable ability (fivefold more) to attach to ATII-like phenotype A549 differentiated cells compared to the *A. baumannii* 17978 parental strain (Figure 5). The results obtained with the ATII-like phenotype A549 cells cultured in Ham’s F-12 Nutrient Mixture for at least 20 days were also confirmed by infecting A549 cells cultured in DMEM with the *A. baumannii* WT or the ΔMac derivative strains (data not shown). When the mutant strain was complemented, the phenotype was restored. The observed increase of bacterial adherence to epithelial cells when *macAB-tolC* was deleted is presumably due to the fact that this strain produced more eDNA that is a cell–cell interconnecting compound (Whitchurch et al., 2002) and overexpressed the *A1S_0112-A1S_0119* operon contributing to the interaction with eukaryotic cells (Rumbo-Feal et al., 2017).

## 4. Conclusion

Even though a positive correlation between biofilm formation and antimicrobial resistance is still debated, clinical *A. baumannii* strains presenting concomitantly a biofilm-forming capacity and a multidrug resistance are currently isolated (Badave and Kulkarni, 2015; Qi et al., 2016; Wang et al., 2018). Indeed, increasing evidence demonstrated that efflux pumps are key actors of antibiotic resistance and also play a role in biofilm formation. They could efflux QS or quorum quenching molecules, as well as EPSs, but also harmful accumulated molecules and can thus promote or regulate biofilm formation (Alav et al., 2018). In *A. baumannii*, MFS and RND-efflux pumps may participate in eDNA release, transport of autoinducer molecules, or adhesion process, but this involvement was suggested to be strain-dependent (Sahu et al., 2012; He et al., 2015; Yoon et al., 2015; Richmond et al., 2016; Lin et al., 2020). Here, we demonstrated that the MacAB-TolC pump is commonly overexpressed in mature S-L biofilms of SDF and ATCC17978 strains. This system, being involved in osmotic protection and probably maintenance of iron homeostasis, may help *A. baumannii* not only to face deleterious conditions present in mature biofilms, where severe ionic gradients can develop. It could help to detoxify cell to persist and to fit in harsh environments, even though the precise substrates of this pump remain to be characterized. Its role in cell wall maintenance demonstrated here is in agreement with its overexpression when bacteria are facing membrane-targeted antibiotics such as colistin (Henry et al., 2014). Shedding some light on the roles of efflux pumps in the biofilm formation may help to develop therapeutic strategies and to improve treatments of biofilm-related infections as well. Design of broad-spectrum and safe efflux pump inhibitors would be valuable tools to both decrease the bacterial biofilm development and restore the activity of antimicrobials.

## Data Availability Statement

The datasets presented in this study can be found in online repositories. The names of the repositories and accession number(s) can be found in the “Materials and Methods” section.

## Author Contributions

AP, VP, and ED contributed to conception and design of the study. MN, BR, HL, AT, AS, J-BV, DV, OL, and VP performed the experiments. MN, BR, HL, AT, TJ, AP, VP, and ED wrote the article. All authors approved the submitted version.

## Conflict of Interest

The authors declare that the research was conducted in the absence of any commercial or financial relationships that could be construed as a potential conflict of interest.

## Publisher’s Note

All claims expressed in this article are solely those of the authors and do not necessarily represent those of their affiliated organizations, or those of the publisher, the editors and the reviewers. Any product that may be evaluated in this article, or claim that may be made by its manufacturer, is not guaranteed or endorsed by the publisher.
