# Supplementary figures and images for: MacAB-TolC Contributes to the Development of Acinetobacter baumannii Biofilm at the Solid–Liquid Interface
Source: Front Microbiol. 2022 Jan 13;12:785161. doi: 10.3389/fmicb.2021.785161 (PMC8792954; doi:10.3389/fmicb.2021.785161)

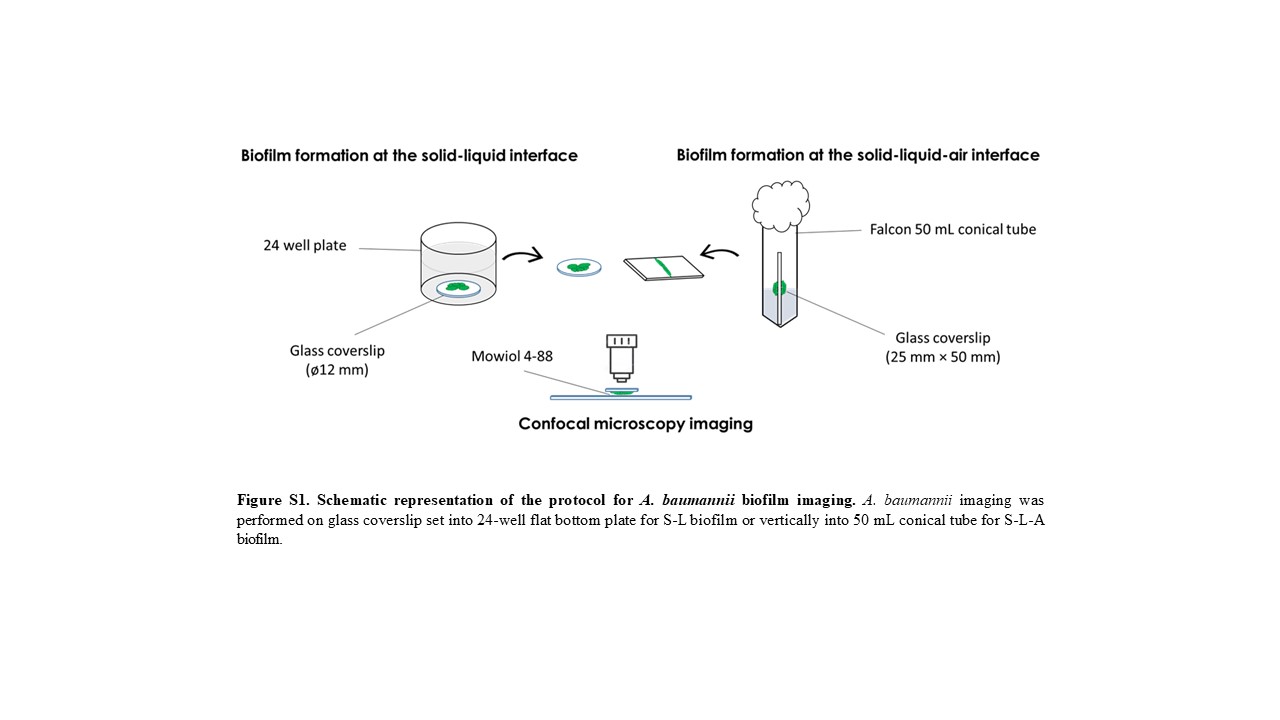

Supplement: Supplementary file 1 [file Image_1.JPEG]

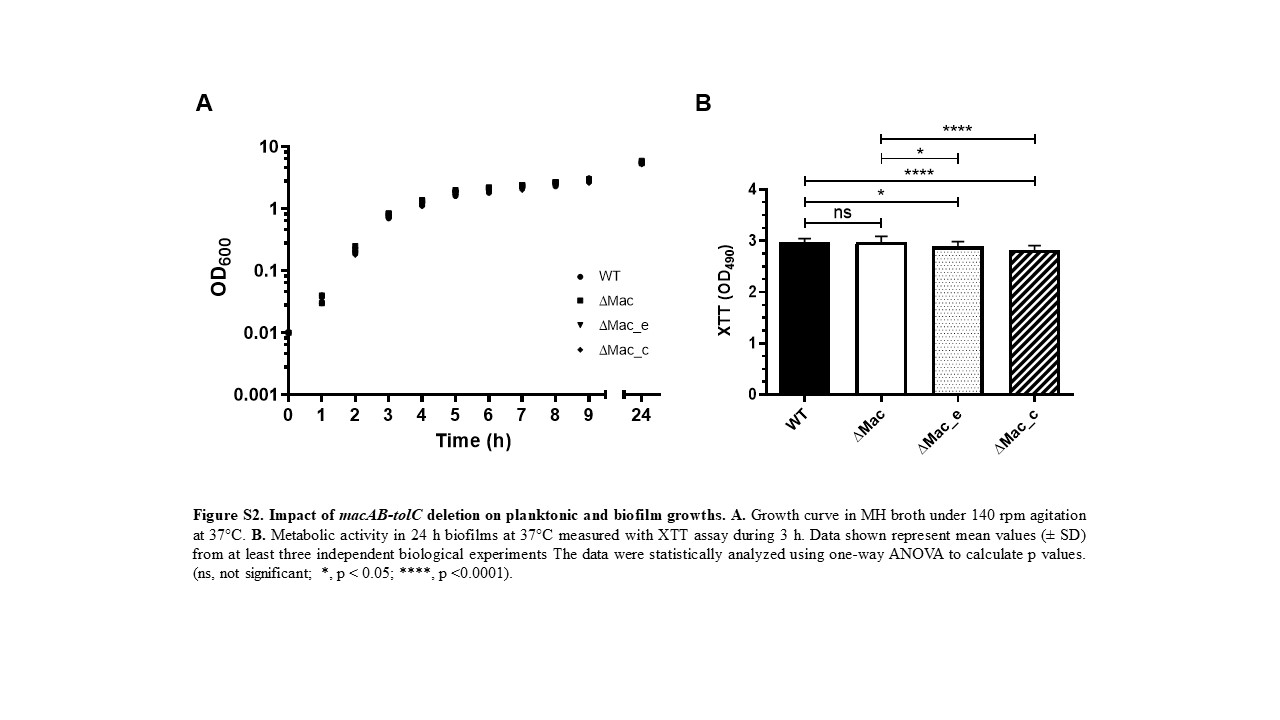

Supplement: Supplementary file 2 [file Image_2.JPEG]

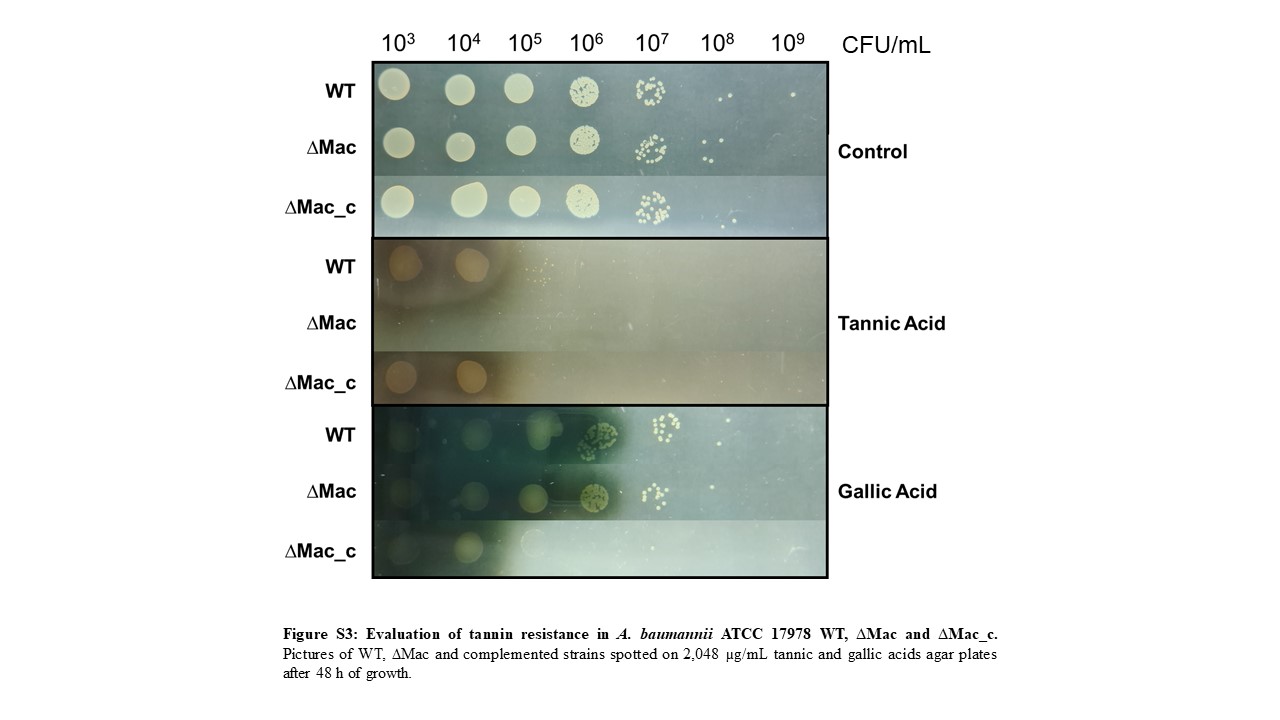

Supplement: Supplementary file 3 [file Image_3.JPEG]

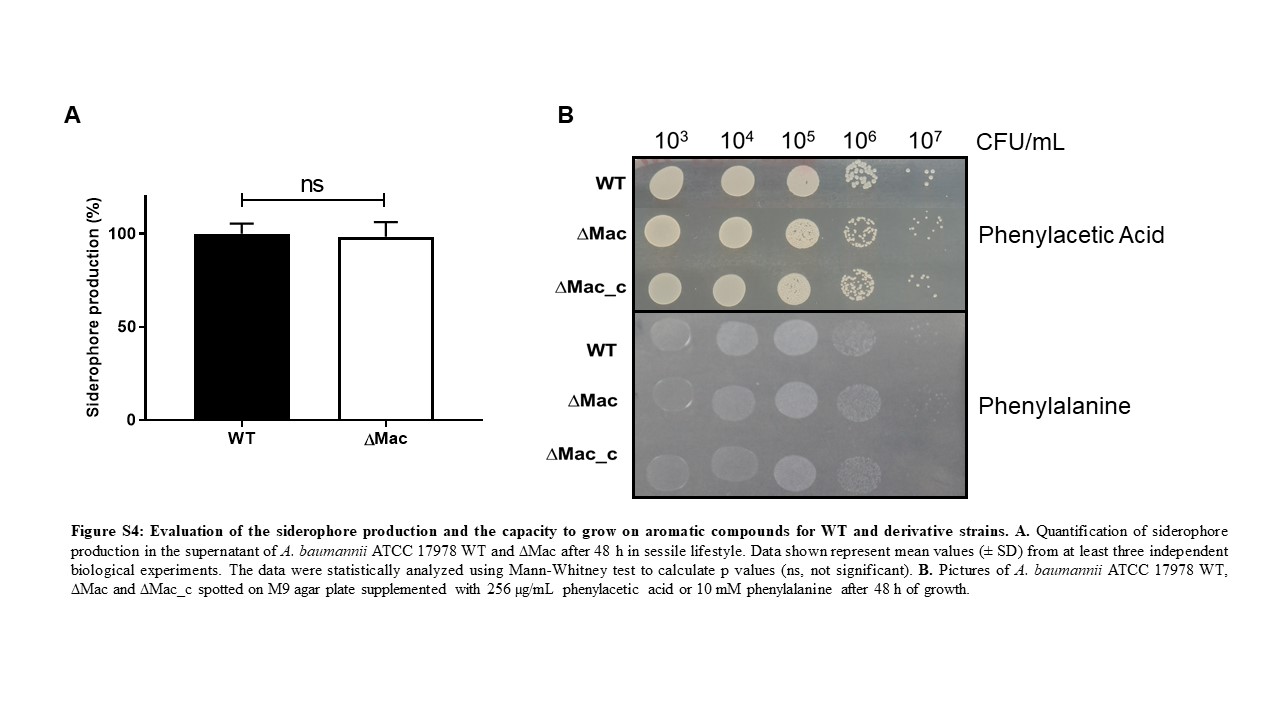

Supplement: Supplementary file 4 [file Image_4.JPEG]
